# CircCAMSAP1 promotes non-small cell lung cancer proliferation and inhibits cell apoptosis by sponging miR-1182 and regulating BIRC5

**DOI:** 10.1080/21655979.2021.2011639

**Published:** 2022-02-08

**Authors:** Yunfei Wang, Xiaobo Li, Huaqi Wang, Guojun Zhang

**Affiliations:** Department of Respiratory and Critical Care Medicine, The First Affiliated Hospital of Zhengzhou University, Zhengzhou, Henan, China

**Keywords:** NSCLC, circCAMSAP1, proliferation, apoptosis, miR-1182

## Abstract

Recently, various studies have suggested that circular RNAs (circRNAs) are ubiquitous in various malignant events, including non-small cell lung cancer (NSCLC) and are closely related to cell proliferation and apoptosis. Unfortunately, the molecular functions involved in this action still have little overlap. Therefore, this study aimed to identify a novel circCAMSAP1 role in NSCLC. Overexpression of circCAMSAP1 has been demonstrated in NSCLC lung tissues and cell lines. Sequencing and RNase R experiments were planned to determine whether circCAMSAP1 is looped and exists in NSCLC. We also found that downregulated circCAMSAP1 repressed cell proliferation and increased apoptosis of NSCLC cells *in vitro* and suppressed xenograft tumor growth *in vivo*. Furthermore, a luciferase assay revealed that circCAMSAP1 could regulate baculoviral inhibitor of apoptosis protein (IAP) repeat containing 5 (BIRC5, also known as survivin) expression by directly binding to miR-1182. However, BIRC5 without 3ʹ untranslated regions (3ʹUTR) could reverse the influence of downregulated circCAMSAP1 on proliferation and apoptosis in NSCLC. Together, our findings reveal a novel mechanism by which the circCAMSAP1/miR-1182/BIRC5 axis promotes NSCLC progression.

## 1. Introduction

As the most common malignant tumor worldwide, lung cancer is an important cause of death [1,2]. Non-small cell lung cancer (NSCLC), which accounts for approximately 85% of lung cancers, consists of diverse categories, such as adenocarcinomas, squamous cell cancers, and large-cell lung cancer [3]. The development of NSCLC is linked to smoking and some environmental factors, such as air pollution; more importantly, recent studies have also shown that the etiopathogenesis of NSCLC is related to abnormal gene mutations [4,5]. Traditional treatment methods mainly include surgery, radiotherapy, and chemotherapy; however, the curative effect is inadequate in those with advanced disease owing to difficulty in diagnosis due to the lack of typical clinical manifestations [6]. Over the last two decades, with a deep understanding of tumor biology, targeted therapy and immunotherapy have greatly improved the clinical outcomes of NSCLC [7]. Therefore, the discovery of new biomarkers and therapeutic targets for patients who do not have sensitive mutations or target gene resistance mutations during targeted therapy is of vital importance.

In recent years, with the rapid development of high-throughput RNA sequencing technology and novel bioinformatics analyses, various circular RNAs (circRNAs) have been examined [8]. It is reported that circRNA, a single-stranded covalently closed continuous loop without the 5ʹ to 3ʹ polarity and polyadenylated tail, is produced by back-splicing progress from precursor messenger RNA (pre-mRNA) and differs from linear RNA [9]. Therefore, circRNA has more stable properties, making it more resistant to RNase R digestion [10]. Growing evidence suggests that circRNAs possess binding sites for microRNAs (miRNAs) and can regulate miRNAs from binding to their targets by acting as miRNA sponges, so that they can regulate various cellular events, including proliferation, apoptosis, and invasion [11,12]. For instance, abnormal expression of circRNA_103993 in NSCLC might be associated with proliferation and apoptosis through the miR-1271/ERG pathway [13]. circRNA AFF4 has been identified in osteoblast cells and may promote tumor progression and inhibit apoptosis [14]. Similarly, circ_0014130 exists in NSCLC, which can also increase cell proliferation and inhibit cell apoptosis [15]. Nevertheless, only a small fraction of the molecular functions of circRNAs in NSCLC have been identified. Thus, our research is devoted to exploring a novel clue for the identification of the underlying role of circRNA in NSCLC.

Thus, we hypothesized that circCAMSAP1 promotes NSCLC proliferation and inhibits cell apoptosis by sponging miR-1182 to increase BIRC5 expression. Simultaneously, the aims and goals of our study were to investigate the abnormal expression and the effect of circCAMSAP1 in NSCLC via *in vitro* and *vivo* experiments. Broadly, by doing so, we may have the opportunity to discover a potential diagnosis and therapeutic target for NSCLC.

## 2. Materials and methods

### 2.1 Clinical specimens

Tissue samples, containing 28 paired NSCLC and corresponding paracancerous tissues, were collected from patients who did not receive chemotherapy or radiotherapy prior to surgical treatment at the First Affiliated Hospital of Zhengzhou University between January 2020 and October 2020. The samples were immediately stored in liquid nitrogen after resection, until the experiments began. Informed consent was obtained from all the participants, and the Ethics Committee of our hospital approved this protocol.

### 2.2 Microarray analysis

Three pairs of NSCLC and adjacent normal tissues were used for circRNA microarray analysis performed by Sinotech Genomics Ltd. (Shanghai, China) to investigate the abnormal expression of circRNAs and select candidate circRNAs [16]. For further details, please refer to the guidelines provided by the company.

### 2.3 Cell lines and cell culture

NSCLC cell lines (A549, H520, H1299, and H460) and normal human bronchial epithelial (NHBE) cells used in our experiments were purchased from the Shanghai Institute of Cell Biology, Chinese Academy of Sciences (Shanghai, China). Cells were incubated in DMEM (Gibco, Grand Island, NY, USA) supplemented with 10% fetal bovine serum (Gibco) and penicillin/streptomycin (100 µg/mL; Gibco) at 37°C with 5% CO_2_.

### 2.4 Cell transfection

The recombinant lentivirus vector sh-circ (to silence circCAMSAP1 expression) and NC (negative control) were constructed and packaged by Gene Pharma (Shanghai, China). The miR-1182 mimic and miR-NC (negative control) were purchased from Solarbio (Beijing, China). pcDNA3.1‐BIRC5 (a vector lacking the BIRC5 3ʹ-UTR) was obtained from Gene Pharma. The transfection assay was performed using Lipofectamine 2000 (Invitrogen, Carlsbad, CA, USA).

### 2.5 RNA extraction, quantitative Real Time-PCR (qRT-PCR), and RNase R assay

Trizol reagent (Invitrogen) was used to isolate total RNA from NSCLC cells. Reverse transcription was performed to obtain cDNAs from RNAs using the TonkBio RT reagent kit (Toneker Biotech, Shanghai, China). Next, qRT-PCR was performed using SYBR Green PCR Master Mix (Solarbio) in an ABI 7500 thermocycler (Thermo Fisher Scientific, Waltham, MA, USA) according to the manufacturer’s instructions. GAPDH and U6 served as the internal controls. Finally, relative expression levels were calculated using 2 ^−ΔΔCt^. Specific primers were synthesized by Tsingke Biotechnology (Beijing, China), and the sequences are presented below.

circCAMSAP1 amplification primers:

Forward primer (F): 5′-ACGTTCAGTGCCTCGAAAGA-3′, Reverse primer (R): 5′-TGTGCTCCTGCTCATACTGG-3′; miR-1182 amplification primers were: Forward primer (F): 5′-GAGGGTCTTGGGAGGGA-3′, Reverse primer (R): 5′-TGGTGTCGTGGAGTCG-3′; GAPDH: Forward primer (F): 5′‐GGGAAACTGTGGCGTGAT‐3′, Reverse primer (R): 5′‐GGGTGTCGCTGTTGAAGT‐3′;

U6: Forward primer (F): 5′‐CTCGCTTCGGCAGCACA‐3′,

Reverse primer (R): 5′‐AACGCTTCACGAATTTGCGT‐3′.

RNase R (Epicenter Biotechnologies, Beijing, China) and RNase R-free water were added to the circCAMSAP1 and line gene groups, respectively, and the relative expression was analyzed using qRT-PCR [17,18].

### 2.6 Cell counting kit (CCK-8) assay

The cells were harvested and incubated in a 96-well plate at 5 × 10^3^ cells per well after being transferred to sh-circ and sh-NC for 48 h. Cell proliferation was determined using a CCK-8 kit (Dojindo; Kumamoto, Japan) after 0, 24, 48, and 72 h of incubation, and a microplate reader (Model ELX800, BioTek, Vermont, USA) was used to record the absorbance (OD) value at 450 nm according to the manufacturer’s protocol. All cells were seeded in three replicate wells [19].

### 2.7 5-Ethynyl-2’-deoxyuridine (EdU) assay

Cell proliferation was also detected using an EdU assay kit (RiboBio, Guangzhou, China). Transfected cells (1 × 10^4^/well) were cultured in a 96-well plate and treated with 100 μL of medium containing 50 μM EdU solution at 37°C for 2 h. The cells were then decolorized with 1 × Apollo® reaction cocktail (100 µL) for 30 min in the dark and washed three times with PBS after fixation with 4% paraformaldehyde solution for 30 min. After that, the nuclei were stained with 100 µL of Hoechst 33,342 and pictures were acquired randomly with a fluorescent microscope (Nikon, Japan) [20].

### 2.8 Flow cytometry (FCM) assay

Forty-eight hours following transfer, cells were harvested by trypsin digestion and washed with PBS. The cells were resuspended in 400 μL of 1× binding buffer to a final concentration of approximately 2 × 10^5^ cells/mL. The apoptotic and dead cells were immediately stained with 5 μL Annexin V-FITC and 10 μL PI at 4°C for 15 min in the dark. Finally, the sum of early and late apoptotic cells was calculated using the following formula: Annexin V+/PI- and Annexin V+/PI+. A flow cytometer (BD Biosciences, San Jose, CA) was used at an excitation and emission wavelength of 488 nm and 539 nm, respectively, following the manufacturer’s instructions, to analyze the apoptotic cells [21].

### 2.9 TUNEL assay

The cell apoptosis rate was also determined using a TUNEL apoptosis assay kit (Yeasen Biotechnology, Shanghai, China). After 48 h of incubation, the transferred cells in 6-well plates were fixed with 4% paraformaldehyde for 30 min. Next, the cells were blocked with 3% H_2_O_2_ for 10 min in the dark after washing three times with PBS. Afterward, the cells were washed and incubated with 0.2% TritonX-100 (100 μL) to increase cell permeability for 5 min at room temperature. Thereafter, 50 μL of TUNEL reaction solution was added to the sample, which was then incubated for 1 h at 37°C in the dark. Finally, the sample was stained with DAPI, which was applied to visualize the cell nuclei, and analyzed using a fluorescent microscope (Nikon, Japan) [22].

### 2.10 Mouse xenograft assay

Ten BALB/c nude mice were obtained from Charles River (Beijing, China); all were female and between 4 and 6 weeks old. The mice were randomly classified into two groups (5 mice/group). These experiments were approved by the Animal Care and Use Committee of Zhengzhou University. We injected 0.1 ml (containing 2 × 10^6^ cells) of cell suspension stably transduced with sh‐circ or sh‐NC subcutaneously into the flanks of nude mice. The tumor volume was measured every 7 days, and data were recorded. Finally, all the mice were sacrificed after 4 weeks, and all lumps were removed and weighed. Tumor tissues were then fixed for hematoxylin and eosin (H&E) or immunohistochemical (IHC) staining [23].

### 2.11 Western blotting assay

Total proteins were extracted using RIPA lysis buffer (Solarbio, Beijing, China), and the concentration was determined using a BCA protein assay kit (Solarbio). The proteins were electrophoresed using sodium dodecyl sulfate-polyacrylamide gel electrophoresis (SDS-PAGE) and transferred to polyvinylidene fluoride (PVDF) membranes. The PVDF membranes were immersed immediately in blocking solution for 2 h on a shaker at room temperature. Next, the samples were incubated overnight on a 4°C shaker after adding the anti-survivin antibody (1:1000, Abcam, UK) and anti-GAPDH antibody (1:1000, Abcam, UK). Thereafter, the membranes were washed three times with TBST on a shaker for 10 min each time and incubated with labeled anti-rabbit secondary antibody (1:5000, Abcam, UK) for 1 h at room temperature. Finally, the bands were visualized using a Bio-Rad image analysis system (Bio-Rad, Hercules, CA, USA). GAPDH served as an internal control [24].

### 2.12 Dual-luciferase reporter assays

The binding sites of circCAMSAP1and miR-1182; miR-1182 and BIRC5 3’-UTR were predicted using https://circinteractome.nia.nih.gov/ and http://www.targetscan.org/. Wild-type and mutated-type circCAMSAP1 or BIRC5 3ʹ-UTR were cloned into pmirGLO vectors (Promega, Madison, WI, USA) after amplification by PCR. 293 T cells were seeded in a 24-well plate at 1 × 10^5^ cells per well overnight before transfection. Luciferase vectors and miR-1182 mimics were co-transfected into 293 T cells using Lipofectamine 2000 (Invitrogen). Ultimately, the results were detected using a dual-luciferase reporter assay system (Promega) and calculated using the formula for Renilla luciferase activity divided by firefly luciferase activity.

### 2.13 Statistical analysis

All statistical analyses were performed using SPSS software (version 21.0; IBM, Armonk, NY, USA). Data are presented as mean ± standard deviation (SD). Comparisons between two or multiple groups were conducted using the Student’s t-test or one-way ANOVA. P < 0.05 indicated significant differences.

## 3. Results

First, the abnormal expression of circCAMSAP1 in NSCLC tissues was detected by microarray analysis and verified in NSCLC tissues and cell lines by qRT-PCR. Therefore, we selected circCAMSAP1 as our gene of interest, and through bioinformatics analysis, we hypothesized that tumor progression could be influenced by the circCAMSAP1/miR-1182/BIRC5 axis. We also designed *in vitro* and *in vivo* experiments to investigate whether circCAMSAP1 promotes NSCLC proliferation and inhibits cell apoptosis. Subsequently, the mechanism was indicated by dual-luciferase reporter and rescue assays, where circCAMSAP1 could activate the malignant process of NSCLC by sponging miR-1182 to increase the expression of BIRC5.

### 3.1 circCAMSAP1 existed and was highly expressed in NSCLC

**Figure 1:**
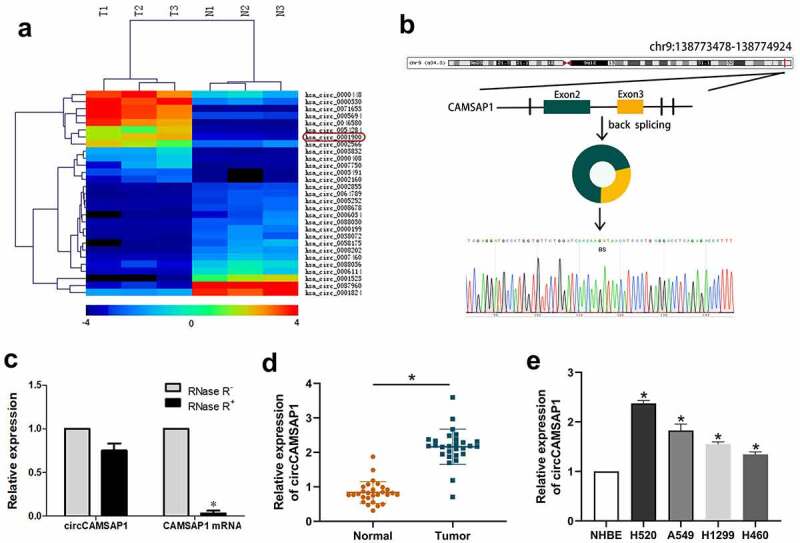
**circCAMSAP1 existed and is highly expressed in NSCLC**. (a) Heatmap: Among three pairs of samples, circCAMSAP1 (also named hsa_circ_0001900) is significantly highly expressed in lung cancer tissues compared with its corresponding paracancerous tissues. (b) Genomic location and splicing mode of circCAMSAP1 show the CAMSAP1 exon 2–3 circularization forming circCAMSAP1. (c) qRT-PCR analysis of circCAMSAP1 and CAMSAP1 linear mRNA with or without RNase R treatment. (d) Relative expression of circCAMSAP1 in 28 NSCLC tissues compared with nontumor tissues by qRT-PCR. (e) Relative expression of circCAMSAP1 in NSCLC cell lines compared with NHBE cells by qRT-PCR. *P < 0.05.

Initially, microarray sequencing was used to analyze circCAMSAP1 expression in three pairs of cancerous and paracancerous tissues from NSCLC samples. A heatmap was created to show the different expression of circRNAs, and we found that a series of circRNAs were upregulated in NSCLC samples and that the expression level of circCAMSAP1 differed significantly (Figure 1a). Thus, we focused on circCAMSAP1 for subsequent research. circCAMSAP1 (also named hsa_circ_0001900) is a loop structure located on chromosome 9 (chr9:138,773,478–138,774,924) that is composed of exons 2 and 3 of the CAMSAP1 (NM_015447) gene through a back-splicing mechanism. The back-splice junction of circCAMSAP1 was verified by Sanger sequencing (Figure 1b). Additionally, the RNase R assay was designed to show that circCAMSAP1 was more stable than linear CAMSAP1; the results demonstrated that the relative expression displayed no difference in circCAMSAP1 after RNase R digestion compared to linear CAMSAP1 (Figure 1c). Finally, the level of circCAMSAP1 in 28 NSCLC tissue samples compared with the adjacent normal tissue samples and NSCLC cell lines (H520, A549, H1299, and H460) compared with NHBE cells were confirmed using qRT-PCR assays, which suggested that NSCLC tissues and cell lines contained a remarkably higher circCAMSAP1 expression, particularly the H520 and A549 cells (Figure 1d, Figure 1e). To conclude, the above evidence collectively established that circCAMSAP1, with a circular structure, was upregulated in NSCLC.

### 3.2 circCAMSAP1 knockdown could inhibit proliferation and promote apoptosis of NSCLC cells

**Figure 2:**
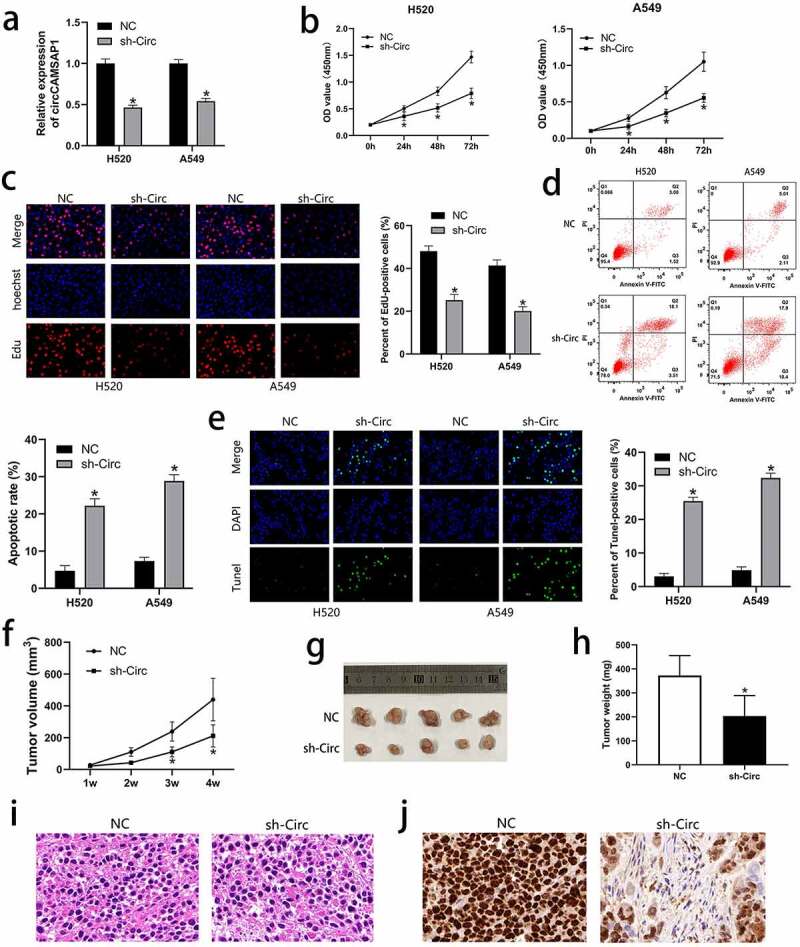
**circCAMSAP1 knockdown could inhibit proliferation and promote apoptosis of NSCLC cells**. (a) circCAMSAP1 expression is downregulated after infection with the recombinant sh‐circ lentivirus in H520 and A549 cells. (b) CCK8 assay indicates that the proliferative capacity of H520 and A549 cells transfected with recombinant sh‐circ lentivirus is substantially decreased relative to that in the control cells. (c) EdU assay was performed to demonstrate that cell proliferation is significantly decreased in cells transfected with recombinant sh‐circ lentivirus compared to cells transfected with NC. (d) FCM assay results show that circCAMSAP1 knockdown increased the apoptotic rate of H520 and A549 cells transfected with recombinant sh‐circ lentivirus. (e) TUNEL assay was conducted to assess cell apoptotic rate after circCAMSAP1 knowdown. (f) Tumor volume was calculated as length × width^2^/2 at the indicated time point. Silencing circCAMSAP1 expression inhibited tumor volume. (g, h) Comparative statistics of tumor imagesand weights of excised tumors at the end point of the xenograft assay. All experiments were repeated at least three times. (i) H&E staining revealed the structure of xenograft tumors between the two groups. (j) IHC staining showed the expression of ki-67 between the two groups. sh‐circ, transfected with recombinant sh‐circ lentivirus NC, negative control. *P < 0.05.

To discuss the biological functions of circCAMSAP1, H520 and A549 cell lines were transfected with the recombinant sh‐circ lentivirus to silence circCAMSAP1. After transfection with sh‐circ, the expression level of circCAMSAP1 was significantly downregulated according to the qRT-PCR results (Figure 2a). We further performed CCK-8 and EdU assays to examine the influence of circCAMSAP1 on the proliferation of NSCLC cells, which showed that circCAMSAP1 silencing inhibited the proliferation ability of H520 and A549 cell lines (Figure 2b, Figure 2c). To determine the influence of circCAMSAP1 expression on cell apoptosis, FCM and TUNEL assays were performed. We found that H520 and A549 cells had a much higher rate of apoptosis after transfection with sh-circ compared to NC (Figure 2d, Figure 2e). These results demonstrated that circCAMSAP1 may be related to NSCLC cell proliferation and apoptosis *in vitro*. Furthermore, a xenograft experiment, containing an sh-circ group (mice injected with the cells infected with sh-circ) and an NC group (mice injected with the cells infected with NC) was used to better indicate tumor growth *in vivo*. The results showed that the tumor volume in the sh-circ group was significantly smaller after 1, 2, 3, and 4 weeks of injection (Figure 2f). Similarly, after euthanizing the mice and calculating the tumor weights, we noticed that circCAMSAP1 knockdown resulted in lighter tumor weights (Figure 2g, Figure 2h). Furthermore, H&E staining showed that circCAMSAP1 knockdown led to smaller cell atypia (Figure 2i). IHC staining showed that the proliferation index ki-67 decreased after downregulating the expression of circCAMSAP1 (Figure 2j). Thus, our findings confirm that circCAMSAP1 plays a crucial role in NSCLC cells by promoting cell proliferation and inhibiting cell apoptosis.

### 3.3 circCAMSAP1 targets and regulates the expression of miR-1182

**Figure 3:**
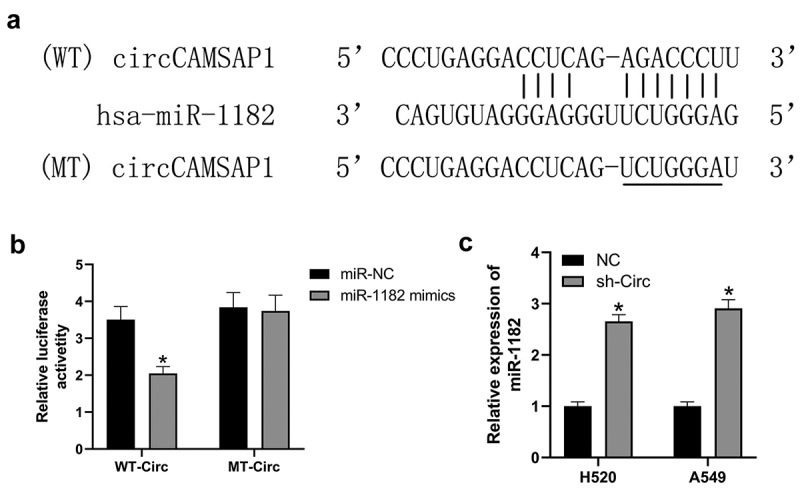
**circCAMSAP1 targets and regulates the expression of miR-1182**. (a) Predicted binding sites of circCAMSAP1 and miR-1182 by bioinformatic analysis. (b) Luciferase reporter assay was performed to validate the direct interaction between circCAMSAP1 and miR-1182. WT‐circ: wild‐type circCAMSAP1 Luciferase reporter plasmids; MT‐circ: mutated‐type circCAMSAP1 Luciferase reporter plasmids. (c) Knockdown of circCAMSAP1 elevated miR-1182 expression in H520 and A549 cells. sh‐circ, transfected with recombinant sh‐circ lentivirus. *P < 0.05.

Recently, numerous investigations have shown that circRNAs can influence gene expression by acting as miRNA sponges [11]. Therefore, we predicted that circCAMSAP1 might bind to miR-1182 by its complementary binding sequences according to bioinformatic analysis (Figure 3a). Next, we designed a dual-luciferase assay to confirm this hypothesis. The relative luciferase activity of the WT-circ group sharply decreased after transfection with miR-1182 mimic compared with miR-NC. While, after transfection with the miR-1182 mimic and miR-NC, the relative luciferase activity of the MT-circ group was similar (Figure 3b). qRT-PCR experiments were designed to further explore whether circCAMSAP1 regulates miR-1182 expression; the results suggested that the level of miR-1182 was markedly increased after silencing circCAMSAP1 in both H520 and A549 cell lines (Figure 3c). Overall, these data revealed that circCAMSAP1 promoted gene expression by sponging miR-1182.

### 3.4 circCAMSAP1 targets BIRC5

**Figure 4:**
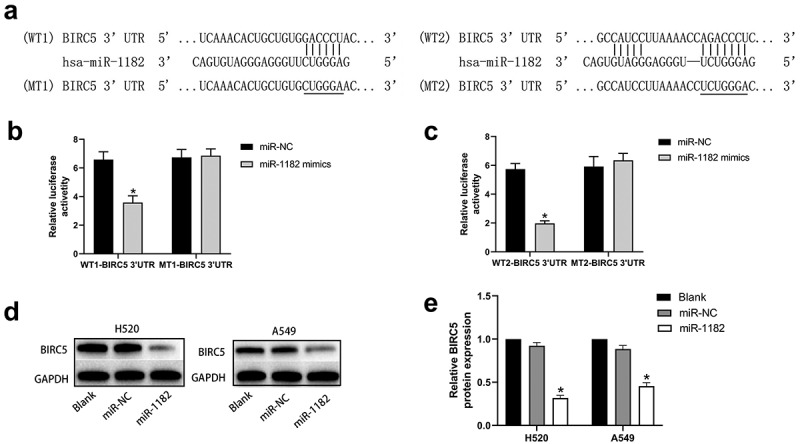
**circCAMSAP1 targets BIRC5**. (a) Predicted binding regions between miR‐1182 and BIRC5 3’-UTR by bioinformatics analysis. (b, c) Luciferase reporter assay was performed to validate the direct interaction between miR-1182 and BIRC5 3’-UTR. WT‐BIRC5 3′‐UTR, wild-type BIRC5 3′‐UTR luciferase reporter plasmids; MT‐BIRC5 3′‐UTR, mutated‐type BIRC5 3′‐UTR luciferase reporter plasmids. (d, e) Western blotting results showed that upregulating miR-1182 expression could significantly decrease BIRC5 protein expression. miR‐1182, cells transfected with miR-1182 mimics; miR‐NC, cells transfected with NC; GAPDH, glyceraldehyde 3‐phosphate dehydrogenase; NC, negative control. *P < 0.05.

Using bioinformatics analysis, we noticed that there were two complementary binding sequences between miR-1182 and BIRC5 3ʹ-UTR (Figure 4a). Thus, BIRC5 may be a potential target of miR-1182. We then performed a dual-luciferase assay to verify our findings. The results demonstrated that the relative luciferase activity in the cells that were co-transfected with WT-BIRC5 3ʹUTR and miR-1182 mimics was considerably lower than that in the control groups. Nevertheless, no significant difference was found in the MT-BIRC5 3ʹ-UTR groups (Figure 4b, Figure 4c). Meanwhile, Western blotting assay was conducted to confirm that upregulation of miR-1182 substantially decreased BIRC5 expression both in H520 and A549 cell lines (Figure 4d, Figure 4e). In general, these assays indicated that miR-1182 targets BIRC5 by binding its 3ʹ-UTR.

### 3.5 BIRC5 upregulation could reverses the effects of circCAMSAP1 silencing and miR-1182 overexpression on proliferation and apoptosis

**Figure 5:**
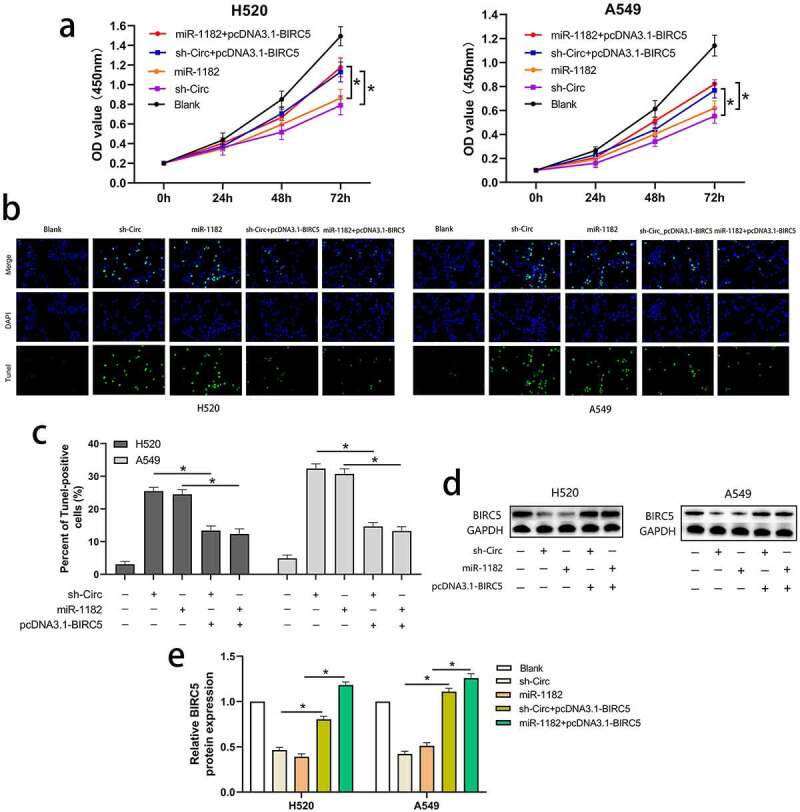
**BIRC5 upregulation could reverse the effects of circCAMSAP1 silencing and miR-1182 overexpression on proliferation and apoptosis**. (a) CCK8 assay indicated the proliferation capacity of cells that were co-transfected with pcDNA3.1‐BIRC5 and sh‐circ; pcDNA3.1‐BIRC5 and miR-1182 was significantly increased relative to cells transfected with sh‐circ and miR-1182 alone. (b, c) TUNEL assay showed the apoptotic rate of cells that were co-transfected with pcDNA3.1‐BIRC5 and sh‐circ; pcDNA3.1‐BIRC5 and miR-1182 was significantly decreased relative to cells transfected with sh‐circ and miR-1182 alone. (d, e) Western blotting assay indicated the BIRC5 expression of cells that were co-transfected with pcDNA3.1‐BIRC5 and sh‐circ; pcDNA3.1‐BIRC5 and miR-1182 was significantly increased relative to cells transfected with sh‐circ and miR-1182 alone. miR‐1182+ pcDNA3.1‐BIRC5, cells co-transfected with miR‐1182 mimics and pcDNA3.1‐BIRC5; sh‐circ+ pcDNA3.1‐BIRC5, cells co-transfected with recombinant sh‐circ lentivirus and pcDNA3.1‐BIRC5; miR‐1182, cells transfected with miR‐1182 mimics; sh‐circ, cells transfected with recombinant sh‐circ lentivirus. *P < 0.05.

To prove that the circCAMSAP1/miR-1182/BIRC5 axis exists in NSCLC, we conducted the following experiments. pcDNA3.1‐BIRC5 (a vector lacking the BIRC5 3′-UTR) and sh-circ were co-transfected into H520 and A549 cell lines to overexpress BIRC5 and silence circCAMSAP1. In addition, we upregulated the expression of BIRC5 and miR-1182 by co-transfecting pcDNA3.1‐BIRC5 and miR-1182 mimics into H520 and A549 cell lines. Furthermore, five groups were divided to examine proliferation and apoptosis using CCK-8 and TUNEL assays. CCK-8 assays showed that compared with sh-circ and miR-1182 mimics groups, the proliferative capacity in H520 and A549 cell lines was notably increased in the groups that were co-transfected with pcDNA3.1‐BIRC5 and sh-circ as well as pcDNA3.1‐BIRC5 and miR-1182 mimics, highlighting that BIRC5 upregulation can reverse the effects of circCAMSAP1 knockdown and miR-1182 overexpression on cell proliferation (Figure 5a). Moreover, the apoptotic capacity was also rescued by overexpressing BIRC5 in TUNEL assays (Figure 5b, Figure 5c). Finally, the protein level of BIRC5 was also detected by Western blotting (Figure 5d, Figure 5e). Together, these results demonstrate that circCAMSAP1 may promote the malignant process of NSCLC via the circCAMSAP1/miR-1182/BIRC5 axis.

## 4. Discussion

circRNAs are a novel type of non-coding RNA that are overwhelmingly generated from back-spliced exons [25]. Emerging evidence has revealed that circRNAs can participate in the malignant progression of various tumor cells by sponging miRNAs to modulate the expression of messenger RNA (mRNA) at the transcriptional or post-transcriptional level [26,27]. As reported by various studies, circRNAs play an indispensable role in the incidence and development of many cancers; therefore, they might be potential molecular markers for diagnosing and treating tumors [28]. Although a number of studies have been conducted to elucidate the molecular mechanism of circRNAs, the research field is still in its infancy [29]. Recent studies have shown that circCAMSAP1 is related to the malignant process of diverse tumors. For example, Luo et al. verified that circCAMSAP1 may be essential in the carcinogenesis of hepatocellular carcinoma by sponging miR-1294 [30]. Chen et al. revealed that circCAMSAP1 promotes proliferation and metastasis of osteosarcoma cells [31]. Zhou et al. reported that circCAMSAP1 has a similar function in colorectal cancer [32]. Nevertheless, this study is the first to demonstrate the biological function of circCAMSAP1 in NSCLC. Here, using bioinformatics analysis, we assumed that there exists a circCAMSAP1/miR-1182/BIRC5 axis in NSCLC.

Subsequently, we conducted a series of experiments to confirm the existence of the circCAMSAP1/miR-1182/BIRC5 axis in NSCLC. First, we designed certain experiments to clarify that circCAMSAP1 is looped and exists in NSCLC. Next, we performed microarray analysis and qRT-PCR assays to demonstrate that circCAMSAP1 expression was much higher in NSCLC tissues and cell lines. In addition, a series of experiments, such as CCK-8, EdU, FCM, TUNEL, and mouse xenograft assays, was performed to elucidate whether circCAMSAP1 had a role in NSCLC proliferation and apoptosis. Meanwhile, a dual-luciferase assay confirmed that circCAMSAP1 sponges miR-1182 via its complementary sequences.

Recent studies have shown that miRNAs play a core role in cell proliferation, survival, and apoptosis by directly sponging their complementary target mRNAs [33]. Li et al. reported that circFMN2 aggravated colorectal cancer tumorigenesis by binding to miR-1182 to stimulate hTERT expression [34]. Hou et al. reported that miR-1182 expression in ovarian cancer was reduced; thus, a possible function of miR-1182 may be promoting malignant tumor behaviors [35]. Huang et al. revealed that circABCC4 promoted the development of prostate cancer by increasing FOXP4 expression by targeting miR‐1182 [36]. In our study, miR-1182 expression was significantly increased in NSCLC after inhibiting the expression of circCAMSAP1. Combined with the dual-luciferase assay, it was verified that circCAMSAP1 might act as a competing endogenous RNA (ceRNA) of miR-1182 to influence NSCLC malignant progression.

BIRC5 is a promising binding target of miR-1182 by bioinformatic analysis, which was examined using a dual-luciferase assay. BIRC5 belongs to the inhibitor of apoptosis protein (IAP) family and is highly expressed in various tumor tissues [37]. Only a few study results have demonstrated that it can influence tumor progression by promoting cell proliferation and inhibiting cell apoptosis since its first discovery in 1997 [38]. Accumulating evidence have also shown that BIRC5 is strongly expressed in tumor cells and may be essential for cell proliferation, apoptosis, and chemotherapy resistance [39]. For example, Zhang et al. reported that elevated levels of BIRC5 can promote the tumorigenesis of hepatocellular carcinoma [40]. Wang et al. also reported that miR-203 suppresses ovarian cancer metastasis by binding to BIRC5 [41]. Zhang et al. showed that BIRC5 exacerbates the development of renal cell carcinoma via the MALAT1/miR-203/BIRC5 axis [42]. Ultimately, we observed that the effect of circCAMSAP1 downregulation and miR-1182 upregulation on cell proliferation and apoptosis could be partly rescued, clarifying that the circCAMSAP1/miR-1182/BIRC5 axis does exist in NSCLC.

The current study focused on exploring the mechanism of NSCLC development. Glycolysis, also known as the Warburg effect, is an important feature of tumor cell metabolism. It can provide ATP for tumor cell proliferation, invasion, and metastasis, thereby promoting the malignant progression of diverse tumors, including NSCLC [43]. Ubiquitination is one of the important pathways of protein degradation. A large amount of evidence shows that members of the IAP family regulate related proteins and signal pathways through the ubiquitinated proteasome system and are closely related to the occurrence and development of a variety of tumors [44]. For instance, circDCUN1D4 could influence the progression of lung adenocarcinoma by suppressing tumor glycolysis [45]. Similarly, ubiquitination is closely related to the progression of retinoblastoma [46]. Therefore, the circCAMSAP1/miR-1182/BIRC5 axis might play an important role in the progression of glycolysis and ubiquitination in NSCLC.

## 5 Conclusion

Collectively, this research corroborated the existence of circCAMSAP1 in NSCLC. Moreover, circCAMSAP1 was overexpressed in NSCLC and could function as a ceRNA to increase cell proliferation and inhibit cell apoptosis via the circCAMSAP1/miR-1182/BIRC5 axis. From these results, a potential therapeutic target may be found in the future.

## Highlights

- circRNAs play a role in malignant events in non-small cell lung cancer (NSCLC)
- circCAMSAP1 overexpression was detected in NSCLC
- Downregulated circCAMSAP1 increased apoptosis and reduced proliferation
- circCAMSAP1/miR-1182/BIRC5 axis promotes NSCLC progression

## References

[1] Siegel RL, Miller KD, Fuchs HE, et al. Cancer statistics, 2021. CA Cancer J Clin. 2021;71(1):7–33. 33433946 10.3322/caac.21654

[2] Chen W, Zheng R, Baade PD, et al. Cancer statistics in China, 2015. CA Cancer J Clin. 2016;66(2):115–132. 26808342 10.3322/caac.21338

[3] Gridelli C, Rossi A, Carbone DP, et al. Non-small-cell lung cancer. Nat Rev Dis Primers. 2015;1(1):15009. 27188576 10.1038/nrdp.2015.9

[4] Musial C, Zaucha R, Kuban-Jankowska A, et al. Plausible role of estrogens in pathogenesis, progression and therapy of lung cancer. Int J Environ Res Public Health. 2021;18(2):648. 10.3390/ijerph18020648 PMC7828659 33466597

[5] Umakanthan S, Bukelo MM. Concise genetic profile of lung carcinoma. Postgrad Med J. 2021. postgradmedj-2021-139860. DOI:10.1136/postgradmedj-2021-139860. 37222713

[6] Duma N, Santana-Davila R, Molina JR. Non-small cell lung cancer: epidemiology, screening, diagnosis, and treatment. Mayo Clin Proc. 2019;94(8):1623–1640. 31378236 10.1016/j.mayocp.2019.01.013

[7] Herbst RS, Morgensztern D, Boshoff C. The biology and management of non-small cell lung cancer. Nature. 2018;553(7689):446–454. 29364287 10.1038/nature25183

[8] Chen B, Huang S. Circular RNA: an emerging non-coding RNA as a regulator and biomarker in cancer. Cancer Lett. 2018;418:41–50. 29330104 10.1016/j.canlet.2018.01.011

[9] Kristensen LS, Andersen MS, Stagsted LVW, et al. The biogenesis, biology and characterization of circular RNAs. Nat Rev Genet. 2019;20(11):675–691. 31395983 10.1038/s41576-019-0158-7

[10] Vo JN, Cieslik M, Zhang Y, et al. The landscape of circular RNA in cancer. Cell. 2019;176(4):869–881. 30735636 10.1016/j.cell.2018.12.021 PMC6601354

[11] Hansen TB, Jensen TI, Clausen BH, et al. Natural RNA circles function as efficient microRNA sponges. Nature. 2013;495(7441):384–388. 23446346 10.1038/nature11993

[12] Wang C, Tan S, Li J, et al. CircRNAs in lung cancer - Biogenesis, function and clinical implication. Cancer Lett. 2020;492:106–115. 32860847 10.1016/j.canlet.2020.08.013

[13] Lv YS, Wang C, Li LX, et al. Effects of circRNA_103993 on the proliferation and apoptosis of NSCLC cells through miR-1271/ERG signaling pathway. Eur Rev Med Pharmacol Sci. 2020;24(16):8384–8393. 32894545 10.26355/eurrev_202008_22635

[14] Mi B, Xiong Y, Chen L, et al. CircRNA AFF4 promotes osteoblast cells proliferation and inhibits apoptosis via the Mir-7223-5p/PIK3R1 axis. Aging (Albany NY). 2019;11(24):11988–12001. 31848327 10.18632/aging.102524 PMC6949079

[15] Wang M, Shi J, Jiang H, et al. Circ_0014130 participates in the proliferation and apoptosis of nonsmall cell lung cancer cells via the miR-142-5p/IGF-1 axis. Cancer Biother Radiopharm. 2020;35(3):233–240. 31916848 10.1089/cbr.2019.2965

[16] Hu W, Han Q, Zhao L, et al. Circular RNA circRNA_15698 aggravates the extracellular matrix of diabetic nephropathy mesangial cells via miR-185/TGF-β1. J Cell Physiol. 2019;234(2):1469–1476. 30054916 10.1002/jcp.26959

[17] Wang Y, Xu R, Zhang D, et al. Circ-ZKSCAN1 regulates FAM83A expression and inactivates MAPK signaling by targeting miR-330-5p to promote non-small cell lung cancer progression. Transl Lung Cancer Res. 2019;8(6):862–875. 32010565 10.21037/tlcr.2019.11.04 PMC6976350

[18] Zhang ZY, Gao XH, Ma MY, et al. CircRNA_101237 promotes NSCLC progression via the miRNA-490-3p/MAPK1 axis. Sci Rep. 2020;10(1):9024. 32494004 10.1038/s41598-020-65920-2 PMC7270109

[19] Hong W, Xue M, Jiang J, et al. Circular RNA circ-CPA4/ let-7 miRNA/PD-L1 axis regulates cell growth, stemness, drug resistance and immune evasion in non-small cell lung cancer (NSCLC). J Exp Clin Cancer Res. 2020;39(1):149. 32746878 10.1186/s13046-020-01648-1 PMC7397626

[20] Yuan C, Yang L. Long non-coding RNA PITPNA-AS1 accelerates the progression of colorectal cancer through miR-129-5p/HMGB1 axis. Cancer Manag Res. 2020;12:12497–12507. 33312000 10.2147/CMAR.S267844 PMC7725105

[21] Yuan Y, Zhou X, Kang Y, et al. Circ-CCS is identified as a cancer-promoting circRNA in lung cancer partly by regulating the miR-383/E2F7 axis. Life Sci. 2021;267:118955. 33359669 10.1016/j.lfs.2020.118955

[22] Li P, Hao L, Guo YY, et al. Chloroquine inhibits autophagy and deteriorates the mitochondrial dysfunction and apoptosis in hypoxic rat neurons. Life Sci. 2018;202:70–77. 29331314 10.1016/j.lfs.2018.01.011

[23] Wu Y, Zhang Y, Zheng X, et al. Circular RNA circCORO1C promotes laryngeal squamous cell carcinoma progression by modulating the let-7c-5p/PBX3 axis. Mol Cancer. 2020;19(1):99. 32487167 10.1186/s12943-020-01215-4 PMC7265647

[24] Ren T, Liu C, Hou J, et al. Hsa_circ_0043265 suppresses proliferation, metastasis, EMT and promotes apoptosis in non-small cell lung cancer through miR-25-3p/FOXP2 pathway. Onco Targets Ther. 2020;13:3867–3880. 32440153 10.2147/OTT.S235231 PMC7213897

[25] Du WW, Zhang C, Yang W, et al. Identifying and characterizing circRNA-protein interaction. Theranostics. 2017;7(17):4183–4191. 29158818 10.7150/thno.21299 PMC5695005

[26] Rong D, Sun H, Li Z, et al. An emerging function of circRNA-miRNAs-mRNA axis in human diseases. Oncotarget. 2017;8(42):73271–73281. 29069868 10.18632/oncotarget.19154 PMC5641211

[27] Zhang HD, Jiang LH, Sun DW, et al. CircRNA: a novel type of biomarker for cancer. Breast Cancer. 2018;25(1):1–7. 28721656 10.1007/s12282-017-0793-9

[28] Lei B, Tian Z, Fan W, et al. Circular RNA: a novel biomarker and therapeutic target for human cancers. Int J Med Sci. 2019;16(2):292–301. 30745810 10.7150/ijms.28047 PMC6367529

[29] Chen LL. The expanding regulatory mechanisms and cellular functions of circular RNAs. Nat Rev Mol Cell Biol. 2020;21(8):475–490. 32366901 10.1038/s41580-020-0243-y

[30] Luo Z, Lu L, Tang Q, et al. CircCAMSAP1 promotes hepatocellular carcinoma progression through miR-1294/GRAMD1A pathway. J Cell Mol Med. 2021;25(8):3793–3802. 33484498 10.1111/jcmm.16254 PMC8051675

[31] Chen Z, Xu W, Zhang D, et al. circCAMSAP1 promotes osteosarcoma progression and metastasis by sponging miR-145-5p and regulating FLI1 expression. Mol Ther Nucleic Acids. 2020;23:1120–1135. 33664993 10.1016/j.omtn.2020.12.013 PMC7901030

[32] Zhou C, Liu HS, Wang FW, et al. circCAMSAP1 promotes tumor growth in colorectal cancer via the miR-328-5p/E2F1 axis. Mol Ther. 2020;28(3):914–928. 31951832 10.1016/j.ymthe.2019.12.008 PMC7054739

[33] Rupaimoole R, Slack FJ. MicroRNA therapeutics: towards a new era for the management of cancer and other diseases. Nat Rev Drug Discov. 2017;16(3):203–222. 28209991 10.1038/nrd.2016.246

[34] Li Y, Li C, Xu R, et al. A novel circFMN2 promotes tumor proliferation in CRC by regulating the miR-1182/hTERT signaling pathways. Clin Sci (Lond). 2019;133(24):2463–2479. 31738400 10.1042/CS20190715

[35] Hou XS, Han CQ, Zhang W. MiR-1182 inhibited metastasis and proliferation of ovarian cancer by targeting hTERT. Eur Rev Med Pharmacol Sci. 2018;22(6):1622–1628. 29630105 10.26355/eurrev_201803_14569

[36] Huang C, Deng H, Wang Y, et al. Circular RNA circABCC4 as the ceRNA of miR-1182 facilitates prostate cancer progression by promoting FOXP4 expression. J Cell Mol Med. 2019;23(9):6112–6119. 31270953 10.1111/jcmm.14477 PMC6714494

[37] Lin TY, Chan HH, Chen SH, et al. BIRC5/Survivin is a novel ATG12-ATG5 conjugate interactor and an autophagy-induced DNA damage suppressor in human cancer and mouse embryonic fibroblast cells. Autophagy. 2020;16(7):1296–1313. 31612776 10.1080/15548627.2019.1671643 PMC7469615

[38] Wheatley SP, Altieri DC. Survivin at a glance. J Cell Sci. 2019;132(7):jcs223826. 30948431 10.1242/jcs.223826 PMC6467487

[39] Kuo HH, Ahmad R, Lee GQ, et al. Anti-apoptotic protein BIRC5 maintains survival of HIV-1-Infected CD4+ T cells. Immunity. 2018;48(6):1183–1194. 29802019 10.1016/j.immuni.2018.04.004 PMC6013384

[40] Zhang M, Yan X, Wen P, et al. CircANKRD52 promotes the tumorigenesis of hepatocellular carcinoma by sponging miR-497-5p and upregulating BIRC5 expression. Cell Transplant. 2021;30:9636897211008874. 33845641 10.1177/09636897211008874 PMC8058805

[41] Wang B, Li X, Zhao G, et al. miR-203 inhibits ovarian tumor metastasis by targeting BIRC5 and attenuating the TGFβ pathway. J Exp Clin Cancer Res. 2018;37(1):235. 30241553 10.1186/s13046-018-0906-0 PMC6150978

[42] Zhang H, Li W, Gu W, et al. MALAT1 accelerates the development and progression of renal cell carcinoma by decreasing the expression of miR-203 and promoting the expression of BIRC5. Cell Prolif. 2019;52(5):e12640. 31250518 10.1111/cpr.12640 PMC6797509

[43] Gao X, Han H. Jolkinolide B inhibits glycolysis by downregulating hexokinase 2 expression through inactivating the Akt/mTOR pathway in non-small cell lung cancer cells. J Cell Biochem. 2018;119(6):4967–4974. 29384225 10.1002/jcb.26742

[44] Mehrotra S, Languino LR, Raskett CM, et al. IAP regulation of metastasis. Cancer Cell. 2010;17(1):53–64. 20129247 10.1016/j.ccr.2009.11.021 PMC2818597

[45] Liang Y, Wang H, Chen B, et al. circDCUN1D4 suppresses tumor metastasis and glycolysis in lung adenocarcinoma by stabilizing TXNIP expression. Mol Ther Nucleic Acids. 2020;23:355–368. 33425493 10.1016/j.omtn.2020.11.012 PMC7779544

[46] Chen X, Chen S, Jiang Z, et al. Ubiquitination-Related miRNA-mRNA interaction is a potential mechanism in the progression of retinoblastoma. Invest Ophthalmol Vis Sci. 2021;62(10):3. 10.1167/iovs.62.10.3 PMC8340667 34347012

